# Deep learning–based assessment of CT markers of sarcopenia and myosteatosis for outcome assessment in patients with advanced pancreatic cancer after high-intensity focused ultrasound treatment

**DOI:** 10.1007/s00330-023-09974-6

**Published:** 2023-08-12

**Authors:** Sebastian Nowak, Christoph Kloth, Maike Theis, Milka Marinova, Ulrike I. Attenberger, Alois M. Sprinkart, Julian A. Luetkens

**Affiliations:** 1 Department of Diagnostic and Interventional Radiology and Quantitative Imaging Lab Bonn (QILaB), University Hospital Bonn, Venusberg-Campus 1, 53127 Bonn, Germany; 2 Department of Nuclear Medicine, University Hospital Bonn, Venusberg-Campus 1, 53127 Bonn, Germany

**Keywords:** Tomography, X-ray computed, Pancreatic carcinoma, Sarcopenia, Survival analysis

## Abstract

**Objectives:**

To evaluate the prognostic value of CT-based markers of sarcopenia and myosteatosis in comparison to the Eastern Cooperative Oncology Group (ECOG) score for survival of patients with advanced pancreatic cancer treated with high-intensity focused ultrasound (HIFU).

**Materials and methods:**

For 142 retrospective patients, the skeletal muscle index (SMI), skeletal muscle radiodensity (SMRD), fatty muscle fraction (FMF), and intermuscular fat fraction (IMFF) were determined on superior mesenteric artery level in pre-interventional CT. Each marker was tested for associations with sex, age, body mass index (BMI), and ECOG. The prognostic value of the markers was examined in Kaplan-Meier analyses with the log-rank test and in uni- and multivariable Cox proportional hazards (CPH) models.

**Results:**

The following significant associations were observed: Male patients had higher BMI and SMI. Patients with lower ECOG had lower BMI and SMI. Patients with BMI lower than 21.8 kg/m^2^ (median) also showed lower SMI and IMFF. Patients younger than 63.3 years (median) were found to have higher SMRD, lower FMF, and lower IMFF. In the Kaplan-Meier analysis, significantly lower survival times were observed in patients with higher ECOG or lower SMI. Increased patient risk was observed for higher ECOG, lower BMI, and lower SMI in univariable CPH analyses for 1-, 2-, and 3-year survival. Multivariable CPH analysis for 1-year survival revealed increased patient risk for higher ECOG, lower SMI, lower IMFF, and higher FMF. In multivariable analysis for 2- and 3-year survival, only ECOG and FMF remained significant.

**Conclusion:**

CT-based markers of sarcopenia and myosteatosis show a prognostic value for assessment of survival in advanced pancreatic cancer patients undergoing HIFU therapy.

**Clinical relevance statement:**

The results indicate a greater role of myosteatosis for additional risk assessment beyond clinical scores, as only FMF was associated with long-term survival in multivariable CPH analyses along ECOG and also showed independence to ECOG in group analysis.

**Key Points:**

*• This study investigates the prognostic value of CT-based markers of sarcopenia and myosteatosis for patients with pancreatic cancer treated with high-intensity focused ultrasound.*

*• Markers for sarcopenia and myosteatosis showed a prognostic value besides clinical assessment of the physical status by the Eastern Cooperative Oncology Group score. In contrast to muscle size measurements, the myosteatosis marker fatty muscle fraction demonstrated independence to the clinical score.*

*• The results indicate that myosteatosis might play a greater role for additional patient risk assessments beyond clinical assessments of physical status.*

## Introduction

Pancreatic cancer is an oncologic disease with a very poor prognosis and an estimated 5-year survival rate of below 10%. Surgical resection can cure pancreatic cancer in early stage; however, the majority of patients are already unresectable at initial diagnosis [1, 2]. Advanced pancreatic cancer is often associated with a very poor quality of life due to cancer pain and a very short life expectancy despite current oncological treatment with chemotherapy or chemoradiotherapy [1]. Local ablation with minimal invasive high-intensity focused ultrasound (HIFU) is an additional treatment option that is often combined with palliative standard treatment, e.g., systemic chemotherapy. With this technique, therapeutic ultrasound (US) waves are focused on the pancreatic lesion to induce coagulative necrosis, leaving healthy tissue outside the focus unharmed. HIFU treatment has been shown to reduce disease-associated symptoms, e.g., cancer pain or tumor mass, and to prolong the survival of patients compared to patients undergoing chemotherapy alone [1, 3, 4].

In patients with pancreatic cancer, a multifactorial syndrome, termed cancer cachexia, is particularly common [5]. This syndrome is induced by reduction of nutritional intake (e.g., due to cancer pain, fatigue, depression, insufficiency of pancreatic enzymes, or side effects of chemotherapy, e.g., nausea and vomiting) and an elevated energy metabolism (e.g., due to increased glucose and protein turnover because of advanced cancer) [2, 6]. Cancer cachexia is associated with ongoing loss of weight, skeletal muscle mass (termed sarcopenia), and physical performance leading to reduced quality of life and life expectancy [2, 5]. An established clinical score for evaluating the physical performance and general physical condition of patients is the Eastern Cooperative Oncology Group (ECOG) performance status that describes how limited the patient is in work activity, self-care, and his walking ability [7]. Evaluating the general physical condition by ECOG showed a strong prognostic value for outcome assessments in pancreatic and other cancer patients [8, 9].

Patients with advanced pancreatic cancer usually receive CT examinations for staging purposes prior to initiation of treatment and during therapy. Besides the diagnostic intention, these CT scans can also be utilized opportunistically to assess the patient’s constitution. Here, muscle size measurements are typically used as a surrogate marker for assessment of muscle wasting in sarcopenia. Additionally, muscle radiodensity evaluations are used to measure infiltration of lipids into the intra- and intermyocellular compartments, termed myosteatosis [10–13]. This opportunistic approach is also driven by recent successes of deep learning in medical imaging, which increases the clinical applicability of image-based analysis by automating otherwise time-consuming tissue segmentations [14–16]. To date, the prognostic value of imaging-based assessment of sarcopenia and myosteatosis has been demonstrated for patients with pancreatic cancer receiving chemotherapy or surgical resection, but not for patients undergoing HIFU therapy [2, 10, 17–19]. For instance, a meta-analysis of multiple studies on body composition and sarcopenia observed significant overall effects for sarcopenia evaluations based on muscle size measurements in CT imaging of patients with resectable and unresectable pancreatic cancer [17]. Another study additionally observed a prognostic value of radiodensity measurements of the muscles in CT for the evaluation of myosteatosis in patients with pancreatic cancer treated with palliative chemotherapy [10].

Therefore, the aim of this study was to evaluate the prognostic value of CT-based assessment of sarcopenia and myosteatosis in comparison to clinical assessment of physical status by ECOG for survival prediction in patients with advanced pancreatic cancer undergoing local US-guided HIFU ablation. Additionally, this study aims to investigate associations between the image-based markers, ECOG, and basic clinical parameters.

## Material and methods

With the approval of institutional review board of the Medical Faculty of the Rheinische Friedrich-Wilhelms-Universitat Bonn, written informed consent was waived due to the retrospective, single-center nature of the study. The study was carried out in compliance with the ethical standards set in the 1964 Declaration of Helsinki as well as its later amendments. Consecutive patients with advanced pancreatic adenocarcinoma undergoing local US-guided HIFU treatment at our center between May 2014 and April 2020 and available CT within 14 days prior to intervention were included. Sex, age, body mass index (BMI), and ECOG were assessed from the clinical data system. The musculus erector spinae was segmented on an axial CT slice at the level of the superior mesenteric artery by a deep learning method [15]. The automatic segmentation was then manually optimized by a medical resident and finally approved by a board-certified radiologist.

### CT-based markers of sarcopenia and myosteatosis

**Fig. 1:**
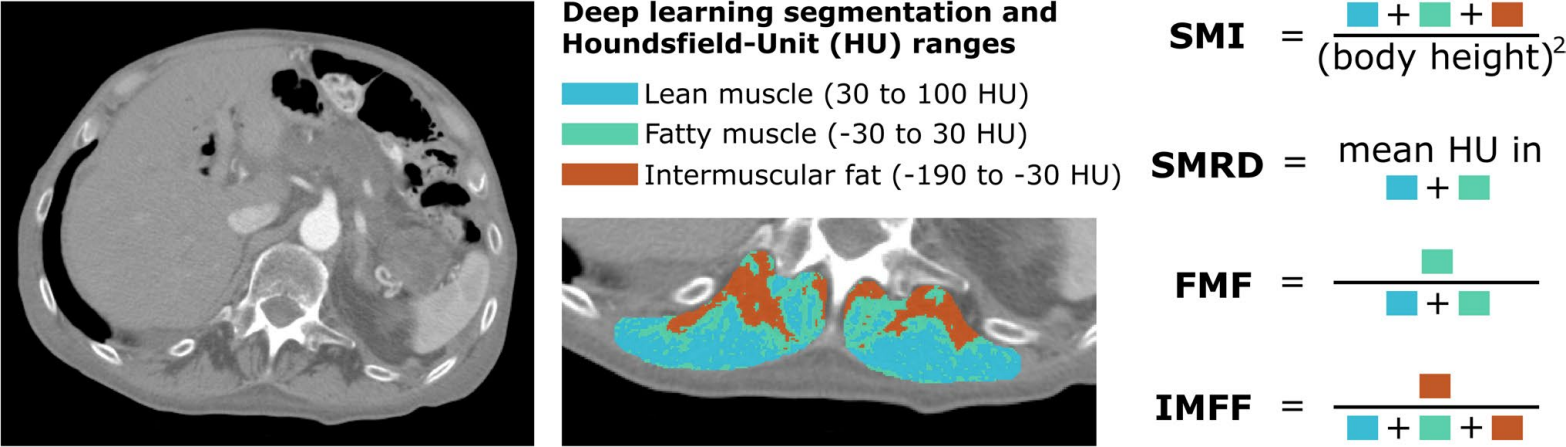
Overview of image-based markers. On the left, a transversal CT scan slice at the level of superior mesenteric artery is shown. The musculus erector spinae was segmented applying a deep learning model. If necessary, automatic segmentations were manually optimized. The segmented area was subdivided into different tissue classes using different ranges of Hounsfield units (HU). The skeletal muscle index (SMI), skeletal muscle radiodensity (SMRD), fatty muscle fraction (FMF), and intermuscular fat fraction (IMFF) were calculated according to the definitions shown on the right

Figure 1 illustrates the computation of image-based markers in detail. To assess muscle size, the established “skeletal muscle index” (SMI) was calculated from the total area of muscle compartment and body height, as frequently applied in muscle-based body composition analysis [2, 10, 12, 18, 20]. For myosteatosis assessment, the two previously proposed markers “skeletal muscle radiodensity” (SMRD) and “fatty muscle fraction” (FMF) were determined [10, 13]. FMF aims to quantify the extent of intramuscular fat infiltration by relating the area of fatty degenerated muscle to the combined area of lean muscle and fatty degenerated muscle. To additionally assess intermuscular fat not captured by SMRD and FMF, the percentage of pure intermuscular fat tissue was extracted as “intermuscular fat fraction” (IMFF).

### Statistical analysis

First, each CT marker was tested for associations with clinical attributes. Therefore, the patients were divided into subgroups for sex, age and BMI (split by the respective median value), ECOG (score 0, 1, and ≥ 2), and the survival status after 1 and 2 years. For each subgroup, median and 25th and 75th interquartile ranges (IQR) are provided. Differences between the subgroups were assessed by the Mann-Whitney *U* test for sex, age, BMI, and survival status, and the Kruskal-Wallis *H* test for ECOG (SciPy 1.8.0) [21]. Differences with *p* value < 0.05 were considered significant.

Then, differences in survival time between subgroups split by clinical and image-based markers were examined in the Kaplan-Meier analysis with the log-rank test. For this, all continuous parameters, i.e., all parameters except sex and ECOG, were divided into subgroups according to sex-specific median values.

Finally, all clinical attributes and imaging markers were examined in univariable CPH models as well as in multivariable CPH models including all parameters. Kaplan-Meier and CPH analyses were conducted with SPSS (27.0.0, IBM).

## Results

Prior to analysis, 153 eligible patients treated with HIFU were identified. Eleven patients were excluded because no CT scan was acquired within 14 days prior to intervention, or due to missing body height or weight records at time of CT imaging. Therefore, a total of 142 patients (73 females, mean age 64.1 ± 10.5 years, range 38–87.5) were included for analysis. Table 1 shows detailed clinical characteristics of the patients included.

**Table 1:** Clinical characteristics of the patients with advanced pancreatic cancer treated by high-intensity focused ultrasound at our center. ECOG:, Eastern Cooperative Oncology Group performance status

	All	Females	Males		All	Females	Males
Site of disease				Biliary drainage			
Body and/or tail	48 (34%)	25 (34%)	23 (33%)	Metallic stent	20 (14%)	11 (16%)	9 (13%)
Head	60 (42%)	28 (38%)	32 (47%)	Plastic stent	14 (10%)	9 (12%)	5 (7%)
Head and body	34 (24%)	20 (28%)	14 (20%)	PTCD	1 (1%)	0 (0%)	1 (1%)
UICC stage				Metastases			
Stage II	2 (1%)	2 (3%)	0 (0%)	Hepatic	65 (46%)	32 (44%)	33 (48%)
Stage III	52 (37%)	26 (36%)	26 (38%)	Pulmonary	12 (8%)	7 (10%)	5 (7%)
Stage IV	83 (58%)	44 (60%)	39 (56%)	Lymph nodes	36 (25%)	18 (25%)	18 (26%)
Recurrence (after Whipple)	5 (4%)	1 (1%)	4 (6%)	Peritoneal	31 (22%)	16 (22%)	15 (22%)
ECOG				Previous treatment			
Status = 0	42 (30%)	15 (21%)	27 (39%)	Chemotherapy	116 (82%)	60 (82%)	56 (81%)
Status = 1	76 (53%)	41 (56%)	35 (51%)	Radiotherapy	9 (6%)	2 (3%)	7 (10%)
Status ≥ 2	24 (17%)	17 (23%)	7 (10%)	Surgery (Whipple)	5 (4%)	1 (1%)	4 (6%)

**Fig. 2:**
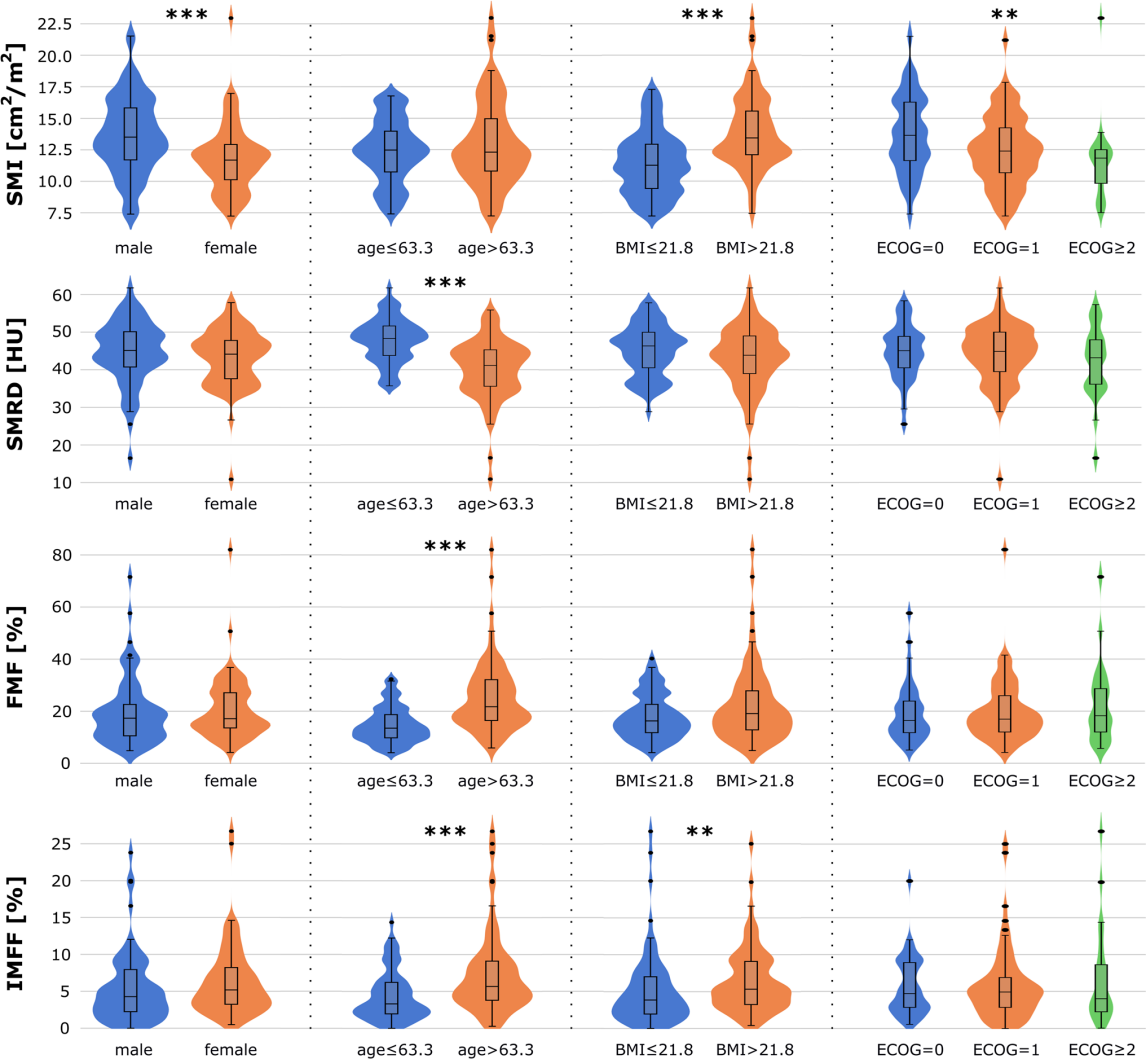
Violin and boxplots for the muscle size assessing the skeletal muscle index (SMI), as well as for myosteatosis assessing markers skeletal muscle radio density (SMRD), fatty muscle fraction (FMF), and intermuscular fat fraction (IMFF) separated for females and males, separated at median age, separated at median body mass index (BMI), and separated for Eastern Cooperative Oncology Group performance status (ECOG). Differences between sexes, age, and BMI were tested using a two-tailed *t* test and between ECOG with Kruskal-Wallis *H* test, with significance indicated by an asterisk (***p values ≤ 0.001, **p values ≤ 0.01, *p values ≤ 0.05)

Median values and interquartile ranges for age, BMI, SMI, SMRD, FMF, and IMFF split into subgroups by sex, median age, median BMI, survival status after 1 and 2 years, and ECOG, are presented in Table 2. The following significant associations were observed: Male patients showed higher BMI and SMI. Patients with lower ECOG score had higher BMI and higher SMI. Patients with BMI higher than 21.8 kg/m^2^ (median) were observed to have higher SMI and higher IMFF. Patients older than 63.3 years (median) showed lower SMRD, higher FMF, and higher IMFF. Patients who survived 1 year had higher BMI and SMI, and patients who survived 2 years had higher BMI compared to patients who died earlier. Figure 2 illustrates violin and boxplots for SMI, BMI, and IMFF split by sex, ECOG score, median age, and median BMI.

**Table 2:** Median values and interquartile ranges of subgroups split by clinical parameters sex, age, body mass index (BMI), and Eastern Cooperative Oncology Group performance status (ECOG) and survival status after 1 and 2 years. Between the investigated subgroups, associations to age, BMI, survival status, skeletal muscle index (SMI), skeletal muscle radiodensity (SMRD), fatty muscle fraction (FMF), and intermuscular fat fraction (IMFF) were tested using the Mann-Whitney *U* test and for ECOG using the Kruskal-Wallis *H* test. Significant differences with *p* value ≤ 0.05 are indicated in bold

Clinical param.	Subgroup	Age	BMI	SMI	SMRD	FMF	IMFF
Sex	Male	63.9 [55.8–72.5]	22.6 [21.3–24.4]	13.5 [11.7–15.8]	45.1 [40.7–50.2]	17.3 [10.5–22.6]	4.3 [2.2–7.9]
	Female	62.1 [56.8–73.2]	20.5 [19.4–22.7]	11.7 [10.1–12.9]	44.1 [37.6–47.8]	17.1 [13.6–27.1]	5.2 [3.3–8.2]
	*p* value	0.64	**< 0.01**	**< 0.01**	0.24	0.4	0.08
Age	> 63.3	-	22.0 [20.0–24.2]	12.3 [10.8–15.0]	41.0 [35.5–45.2]	21.9 [16.8–32.2]	5.8 [3.9–9.1]
	≤ 63.3	-	21.8 [19.8–23.0]	12.5 [10.8–14.2]	48.4 [43.9–51.7]	13.2 [9.7–18.6]	3.3 [1.9–6.2]
	*p* value	-	0.43	0.54	**< 0.01**	**< 0.01**	**< 0.01**
BMI	> 21.8	64.3 [56.6–73.3]	-	13.5 [12.1–15.7]	43.9 [38.7–49.1]	19.1 [12.5–27.9]	5.3 [3.2–9.0]
	≤ 21.8	62.0 [56.6–71.7]	-	11.2 [9.4–12.9]	46.5 [40.6–49.8]	15.8 [11.8–22.5]	3.9 [2.0–7.2]
	*p* value	0.63	-	**< 0.01**	0.17	0.14	**0.01**
ECOG	= 0	61.3 [54.6–67.1]	22.6 [20.8–26.1]	13.7 [11.7–16.3]	45.1 [40.6–48.9]	16.5 [11.8–23.9]	4.7 [2.9–8.9]
	= 1	64.9 [56.7–73.4]	21.6 [19.6–23.2]	12.4 [10.7–14.2]	44.9 [39.5–50.0]	17.0 [12.0–26.0]	5.0 [2.9–6.9]
	≥ 2	62.0 [59.2–74.6]	21.1 [19.4–22.9]	11.9 [9.8–12.5]	43.2 [36.2–48.0]	18.2 [12.0–28.6]	4.0 [2.3–8.7]
	*p* value	0.17	**0.03**	**0.01**	0.47	0.74	0.94
Survival status after 1 year	Died	61.8 [56.6–73.2]	21.5 [19.6–23.4]	12.2 [10.5–13.7]	45.1 [38.8–50.1]	16.8 [11.9–26.7]	4.6 [2.3–7.8]
	Survived	64.3 [55.6–71.6]	22.5 [20.8–25.0]	13.5 [10.9–16.6]	44.1 [39.9–48.8]	18.8 [11.6–25.6]	4.9 [3.0–7.8]
	*p* value	0.96	**0.02**	**0.01**	0.97	0.95	0.65
Survival status after 2 years	Died	62.1 [56.6–73.2]	21.8 [19.8–23.6]	12.3 [10.7–14.2]	45.1 [38.5–50.0]	17.1 [11.8–27.1]	4.9 [2.4–7.9]
	Survived	61.9 [56.1–65.3]	24.4 [22.3–27.9]	14.3 [12.6–15.6]	44.1 [42.7–47.6]	19.5 [14.3–21.2]	4.7 [4.2–5.1]
	*p* value	0.47	**0.02**	0.10	0.83	0.77	0.74

**Fig. 3:**
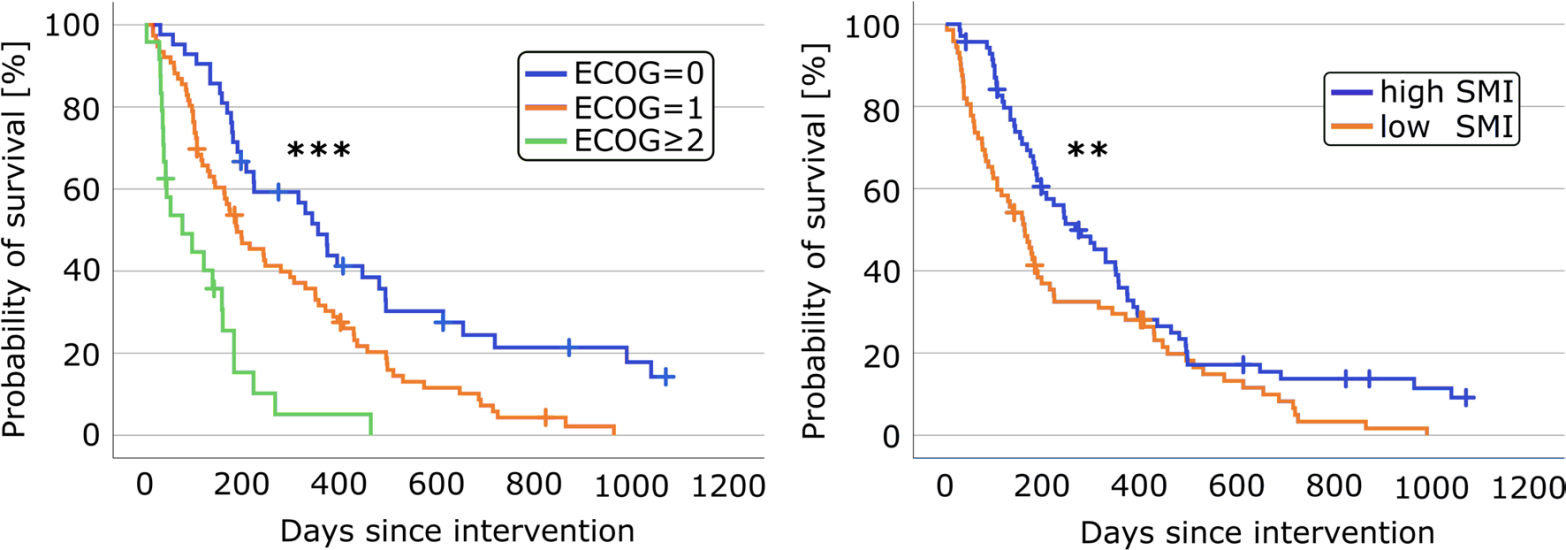
The Kaplan-Meier curves for the 3-year survival of patients separated by the Eastern Cooperative Oncology Group performance status (ECOG) and by sex-specific median of the skeletal muscle index (SMI). Differences between groups were tested by the log-rank test and significance is indicated by an asterisk (***p values ≤ 0.001, **p values ≤ 0.01)

Table 3 shows 1-, 2-, and 3-year survival and the results of the Kaplan-Meier analysis with log-rank test for patients’ subgroups split by clinical and imaging-based parameters. Overall, the median survival time of all patients was 185 (IQR: 99–404) days. Only 10 patients survived longer than 2 years. Patients with low SMI and patients with higher ECOG score had lower survival times. Figure 3 shows the corresponding 3-year Kaplan-Meier survival curves. Hazard ratios and *p* values of the univariable and multivariable CPH models are shown in Table 4. Univariable CPH analyses for 1-, 2-, and 3-year survival showed that higher ECOG score, lower BMI, and lower SMI were associated with increased patient risk. Combining all parameters in multivariable CPH analyses for 1-year survival revealed that higher ECOG score, lower SMI, lower IMFF, and higher FMF were associated with increased patient risk. When parameters are examined in multivariable CPH models for 2- and 3-year survival, only ECOG and FMF remained significant.

**Table 3:** Evaluation of predictors of 1-, 2-, and 3-year survival in patients with advanced pancreatic cancer undergoing ultrasound-guided HIFU treatment using the Kaplan-Meier analysis. Differences in survival times were tested by the log-rank test. For each variable, patients were split into subgroups. For age, body mass index (BMI), skeletal muscle index (SMI), skeletal muscle radiodensity (SMRD), fatty muscle fraction (FMF), and intermuscular fat fraction (IMFF), patients were split according to the sex-specific median (SSM). Median survival times for each subgroup are given with 95% confidence interval. *p* values ≤ 0.05, that indicate significance, are highlighted in bold

			Number of events in…			Median survival	*p* value of the log-rank test		
Variable	Subgroup	*N*	1 year	2 years	3 years	Time [days]	1 year	2 years	3 years
Sex	Male	69	40	60	62	222 [122–322]	0.10	0.23	0.21
	Female	73	53	63	65	185 [161–208]			
Age	≤ SSM	72	51	63	67	188 [138–238]	0.47	0.87	0.69
	> SSM	70	42	60	60	196 [152–240]			
BMI	≤ SSM	73	51	66	68	171 [138–204]	0.18	0.21	0.17
	> SSM	69	42	57	59	241 [120–362]			
ECOG	= 0	42	21	31	33	353 [283–423]	**< 0.01**	**< 0.01**	**< 0.01**
	= 1	76	51	70	72	187 [118–256]			
	≥ 2	24	21	22	22	75 [0–154]			
SMI	≤ SSM	72	50	66	68	161 [116–205]	**0.04**	**0.02**	**0.01**
	> SSM	70	43	57	59	265 [174–356]			
FMF	≤ SSM	72	51	64	66	173 [138–207]	0.26	0.22	0.16
	> SSM	70	42	59	61	206 [152–260]			
SMRD	≤ SSM	72	43	60	62	213 [161–265]	0.23	0.13	0.09
	> SSM	70	50	63	65	175 [140–210]			
IMFF	≤ SSM	72	48	63	64	173 [136–210]	0.49	0.52	0.72
	> SSM	70	45	60	63	222 [132–312]

**Table 4:** Evaluation of predictors of 1-, 2-, and 3-year mortality in patients with pancreatic cancer undergoing high-intensity focused ultrasound therapy using Cox proportional hazards models. First univariable analysis was performed with the imaging-based markers (skeletal muscle index (SMI), skeletal muscle radiodensity (SMRD), fatty muscle fraction (FMF), and intermuscular fat fraction (IMFF)) and clinical attributes (sex, age, body mass index (BMI), Eastern Cooperative Oncology Group performance status (ECOG)). Then, multivariable models with inclusion of all parameters were tested. Significant hazard ratios with *p* values ≤ 0.05 are shown in bold

	1-year survival				2-year survival				3-year survival			
	Univariable analysis		Multivariable analysis		Univariable analysis		Multivariable analysis		Univariable analysis		Multivariable analysis	
Variables	Hazard ratio	*p*	Hazard ratio	*p*	Hazard ratio	*p*	Hazard ratio	*p*	Hazard ratio	*p*	Hazard ratio	*p*
Sex	0.71 [0.47–1.08]	0.11	1.05 [0.66–1.67]	0.85	0.80 [0.56–1.15]	0.23	1.11 [0.75–1.65]	0.61	0.80 [0.56–1.14]	0.22	1.13 [0.76–1.66]	0.55
Age	1.00 [0.98–1.02]	0.88	0.99 [0.97–1.01]	0.37	1.00 [0.99–1.02]	0.73	0.99 [0.97–1.01]	0.46	1.00 [0.98–1.02]	0.88	0.99 [0.97–1.01]	0.34
BMI	0.93 [0.87–0.99]	**0.02**	0.99 [0.92–1.06]	0.70	0.94 [0.89–0.99]	**0.02**	0.97 [0.92–1.03]	0.36	0.94 [0.89–0.99]	**0.01**	0.97 [0.92–1.03]	0.35
ECOG	2.18 [1.57–3.04]	**< 0.01**	2.23 [1.54–3.24]	**< 0.01**	2.15 [1.60–2.90]	**< 0.01**	2.29 [1.64–3.19]	**< 0.01**	2.22 [1.65–2.98]	**< 0.01**	2.36 [1.70–3.28]	**< 0.01**
SMI	0.88 [0.82–0.95]	**< 0.01**	0.90 [0.82–0.98]	**0.02**	0.92 [0.86–0.98]	**0.01**	0.94 [0.87–1.01]	0.09	0.91 [0.86–0.97]	**< 0.01**	0.94 [0.87–1.01]	0.07
SMRD	0.99 [0.97–1.02]	0.67	1.06 [0.97–1.15]	0.24	1.00 [0.97–1.02]	0.68	1.06 [0.98–1.15]	0.13	1.00 [0.97–1.02]	0.76	1.06 [0.98–1.15]	0.14
FMF	1.00 [0.99–1.02]	0.67	1.07 [1.01–1.14]	**0.03**	1.00 [0.99–1.02]	0.56	1.07 [1.01–1.13]	**0.02**	1.00 [0.99–1.02]	0.66	1.07 [1.01–1.13]	**0.02**
IMFF	0.99 [0.94–1.03]	0.57	0.94 [0.89–1.00]	**0.05**	0.99 [0.95–1.03]	0.72	0.96 [0.91–1.01]	0.09	0.99 [0.95–1.03]	0.76	0.96 [0.91–1.01]	0.13

## Discussion

This study investigates the prognostic value of CT imaging markers for sarcopenia and myosteatosis in comparison to clinical assessment of physical status by the ECOG score in patients with advanced pancreatic cancer treated with local US-guided HIFU ablation in addition to other palliative oncological treatments.

The results demonstrate that the ECOG performance status is a strong predictor of patient survival following HIFU therapy in both, Kaplan-Meier and CPH analyses. Previous studies have already demonstrated that the assessment of the patient’s general physical condition by the ECOG score has a strong prognostic value for pancreatic cancer patients treated with chemo- or chemoradiotherapy and for other cancer patients [8, 9]. Interestingly, other previous studies that evaluated ECOG for pancreatic cancer patients treated with chemotherapy and/or surgical resection did not observe such a strong prognostic value [18, 19]. However, only two ECOG groups were considered in these studies (ECOG = 0 and ≥ 1). This indicates that particularly a score of ECOG ≥ 2 may be associated with increased mortality.

We investigated SMI, as this marker already demonstrated a prognostic value for patients with pancreatic cancer treated by surgical resection and for various other oncological diseases in previous studies [17, 19, 20, 22, 23]. In the subgroup analysis, SMI showed a significant correlation to BMI. The significant hazard ratios of BMI and SMI in the univariable CPH analysis, their lower prognostic value in the multivariable CPH analyses, and their observed associations with ECOG suggest that part of the information of BMI and SMI is already well reflected by ECOG. However, the significant differences in SMI between patients who survived and patients who died within 1 year, along with the prognostic value of SMI in Kaplan-Meier and in the multivariable CPH analysis for 1-year survival, indicate that image-based assessment of muscle size on CT imaging prior to HIFU treatment provides additional information particularly for short-term survival.

In addition to quantification of muscle size by SMI, which is typically used for image-based assessment of sarcopenia, we also investigated markers assessing myosteatosis. Of note, in contrast to SMI, all myosteatosis markers were associated with age. The increase of myosteatosis with age was also described in previous studies [11]. The markers for myosteatosis showed no associations with the ECOG performance score, sex nor BMI, while SMI and BMI were associated with sex and ECOG.

Mean radiodensity of the musculature is a myosteatosis marker that showed prognostic relevance within univariable CPH analyses in a previous study of patients with unresectable pancreatic cancer treated with palliative chemotherapy [10]. However, for the patient cohort of our study, no prognostic value of SMRD was observed in Kaplan-Meier nor CPH analyses.

Besides SMI and SMRD, we also investigated the two markers FMF and IMFF explicitly aimed at assessing inter- and intramuscular fat infiltration in myosteatosis. These two markers did not show any prognostic value when considered in Kaplan-Meier or univariable CPH analysis alone. However, both markers were significant predictors along with SMI when combined with clinical parameters in multivariable CPH analysis for 1-year survival. Furthermore, FMF was the only image-based marker that retained predictive value along with ECOG in the multivariable Cox models for 2- and 3-year survival.

Interestingly, IMFF was observed as a protective predictor with hazard ratios below one, in contrast to FMF, for which patient risk increases with higher values. Due to the observed association of the protective predictor IMFF with BMI, it may be assumed that larger intermuscular fat depots represented a better nutritional status that prolongs short-term survival in the current cohort.

As described in other studies, the results of our study also underscore that sarcopenia and myosteatosis are not synonymous and that assessment of myosteatosis has the potential to provide important additional information [11].

## Conclusion

In conclusion, this study demonstrates that image-based markers of sarcopenia and myosteatosis derived from pre-therapeutic CT scans have a prognostic value for patients with advanced pancreatic cancer after palliative HIFU therapy. Image-based assessment of myosteatosis might play a greater role in the evaluation of a patient’s physical status along with the established ECOG score than simple muscle size measurements.

## Abbreviations

BMI: Body mass index
CPH: Cox proportional hazards
ECOG: Eastern Cooperative Oncology Group
FMF: Fatty muscle fraction
HIFU: High-intensity focused ultrasound
IMFF: Intermuscular fat fraction
IQR: Interquartile range
SMI: Skeletal muscle index
SMRD: Skeletal muscle radiodensity
US: Ultrasound

## Declarations

### Guarantor

The scientific guarantor of this publication is PD Dr. med. Julian Luetkens.

### Statistics and biometry

No complex statistical methods were necessary for this paper.

### Study subjects or cohorts overlap

Some study subjects were included in a previous study. The aim of this previous study was to evaluate if a HIFU-induced early sterile inflammatory reaction is initiated after ablation of uterine fibroids and pancreatic carcinoma. The research question of the current submitted study has no overlap to this previous investigation (DOI: 10.1080/02656736.2021.1900926).

### Methodology

- retrospective
- prognostic study
- performed at one institution

## References

[1] Sofuni A, Asai Y, Mukai S, Yamamoto K, Itoi T (2022). High-intensity focused ultrasound therapy for pancreatic cancer. J Med Ultrason..

[2] Naumann P, Eberlein J, Farnia B (2019). Cachectic body composition and inflammatory markers portend a poor prognosis in patients with locally advanced pancreatic cancer treated with chemoradiation. Cancers.

[3] Marinova M, Huxold HC, Henseler J (2019). Clinical effectiveness and potential survival benefit of US-guided high-intensity focused ultrasound therapy in patients with advanced-stage pancreatic cancer. Ultraschall Med.

[4] Marinova M, Feradova H, Gonzalez-Carmona MA (2021). Improving quality of life in pancreatic cancer patients following high-intensity focused ultrasound (HIFU) in two European centers. Eur Radiol.

[5] Dhanapal R, Saraswathi TR, Govind RN (2011). Cancer cachexia. J Oral Maxillofac Pathol.

[6] Fearon KC, Moses AG (2002). Cancer cachexia. Int J Cardiol.

[7] Oken MM, Creech RH, Tormey DC (1982). Toxicity and response criteria of the Eastern Cooperative Oncology Group. Am J Clin Oncol.

[8] Kalser MH, Barkin J, Macintyre JM (1985). Pancreatic cancer. Assessment of prognosis by clinical presentation. Cancer.

[9] Demirelli B, Babacan NA, Ercelep Ö (2021). Modified Glasgow prognostic score, prognostic nutritional index and ECOG performance score predicts survival better than sarcopenia, cachexia and some inflammatory indices in metastatic gastric cancer. Nutr Cancer.

[10] Rollins KE, Tewari N, Ackner A (2016). The impact of sarcopenia and myosteatosis on outcomes of unresectable pancreatic cancer or distal cholangiocarcinoma. Clin Nutr.

[11] Correa-de-Araujo R, Addison O, Miljkovic I (2020). Myosteatosis in the context of skeletal muscle function deficit: an interdisciplinary workshop at the National Institute on Aging. Front Physiol.

[12] Murray TE, Williams D, Lee MJ (2017). Osteoporosis, obesity, and sarcopenia on abdominal CT: a review of epidemiology, diagnostic criteria, and management strategies for the reporting radiologist. Abdom Radiol (NY).

[13] Luetkens JA, Faron A, Geissler HL (2020). Opportunistic computed tomography imaging for the assessment of fatty muscle fraction predicts outcome in patients undergoing transcatheter aortic valve replacement. Circulation.

[14] Magudia K, Bridge CP, Bay CP (2021). Population-scale CT-based body composition analysis of a large outpatient population using deep learning to derive age-, sex-, and race-specific reference curves. Radiology.

[15] Nowak S, Faron A, Luetkens JA (2020). Fully automated segmentation of connective tissue compartments for CT-based body composition analysis: a deep learning approach. Invest Radiol.

[16] Nowak S, Theis M, Wichtmann BD (2022). End-to-end automated body composition analyses with integrated quality control for opportunistic assessment of sarcopenia in CT. Eur Radiol.

[17] Bundred J, Kamarajah SK, Roberts KJ (2019). Body composition assessment and sarcopenia in patients with pancreatic cancer: a systematic review and meta-analysis. HPB (Oxford).

[18] Basile D, Parnofiello A, Vitale MG (2019). The IMPACT study: early loss of skeletal muscle mass in advanced pancreatic cancer patients. J Cachexia Sarcopenia Muscle.

[19] Sugimoto M, Farnell MB, Nagorney DM (2018). Decreased skeletal muscle volume is a predictive factor for poorer survival in patients undergoing surgical resection for pancreatic ductal adenocarcinoma. J Gastrointest Surg.

[20] Faron A, Opheys NS, Nowak S (2021). Deep learning-based body composition analysis predicts outcome in melanoma patients treated with immune checkpoint inhibitors. Diagnostics.

[21] Virtanen P, Gommers R, Oliphant TE (2020). SciPy 10: fundamental algorithms for scientific computing in Python. Nat Methods.

[22] Prado CM, Lieffers JR, McCargar LJ (2008). Prevalence and clinical implications of sarcopenic obesity in patients with solid tumours of the respiratory and gastrointestinal tracts: a population-based study. Lancet Oncol.

[23] Shachar SS, Williams GR, Muss HB, Nishijima TF (2016). Prognostic value of sarcopenia in adults with solid tumours: a meta-analysis and systematic review. Eur J Cancer.

